# Predictors and Prevalence of Sugar‐Sweetened Beverages Consumption Among Health Care Professionals in Tamale, Ghana

**DOI:** 10.1002/puh2.70354

**Published:** 2026-09-10

**Authors:** Michael Akenteng Wiafe, Hamza Sulemana, Gabriel Libienuo Sowley Kalog, Kwadwo Densi Amankwah

**Affiliations:** ^1^ Department of Dietetics University for Development Studies Tamale Ghana; ^2^ Department of Nutritional Sciences University for Development Studies Tamale Ghana; ^3^ Department of Health Science Brunel University of London Uxbridge UK

**Keywords:** health care professionals, predictors, sugar‐sweetened beverages

## Abstract

**Background:**

The study assessed the consumption of sugar‐sweetened beverages (SSBs) and its associated factors among health care professionals in the Tamale Metropolis.

**Methods:**

In this cross‐sectional study, 345 health care professionals were randomly recruited. A semi‐structured questionnaire was used to collect data on sociodemographic, screen time and SSBs. Descriptive, chi‐square and regression analyses were used in the statistical analysis.

**Results:**

About 98% of participants had ever consumed SSBs and frequently consumed were sweetened local juice (61.6%), regular soft drink (50.4%), and sweetened coffee/tea (73.6%). Nurses (OR = 11.42, *p* < 0.05, 95% CI: 2.55–51.22), pharmacists (OR = 6.08, *p* = 0.04, 95% CI: 1.07–34.58) and nutritionists (OR = 5.37, *p* = 0.04, 95% CI: 1.02–28.31) had an increased risk of sweetened local juice intake. The odds of consuming regular soft drinks (OR = 3.27, *p* < 0.05, 95% CI: 1.72–6.21) and sport and energy drink (OR = 3.61, *p* < 0.05, 95% CI: 1.40–9.32) were higher among married participants. Female participants (OR = 4.07, *p* < 0.05, 95% CI: 1.79–9.26) had higher odds of consuming sport and energy drink.

**Conclusion:**

Health care professionals showed a high consumption rate of SSBs and frequently consumed sweetened coffee/tea; however, they had even higher odds of sweetened local juice consumption.

## Graphical abstract

Sugary Drinks Consumption Among Health Care Professionals

Running message:

High SSB consumption was observed among health care professionals. Sweetened coffee/tea was the most frequently consumed beverage, whereas sweetened local juice consumption showed a significant association with professional category.

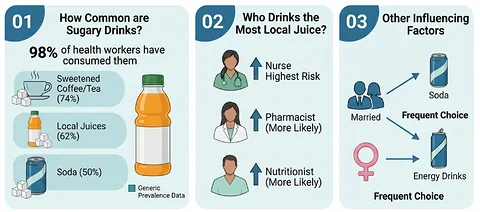

## 1 Background

According to the World Health Organization (WHO), sugar‐sweetened beverages (SSBs) are beverages sweetened with added free sugar or other caloric sweeteners, such as brown sugar, corn sweeteners, corn syrup, dextrose, fructose, glucose, high fructose corn syrup, honey, lactose, malt syrup, maltose, molasses, raw sugar and sucrose [1]. Examples of SSBs include regular soda, vitamin water, flavoured milk, fruit punch, soft drinks, sports drinks, energy drinks, sweetened waters and coffee and tea beverages with added sugar [1, 2, 3].

SSBs and processed or fried foods are commonly sold in formal outlets and are often advertised through internet and television in Ghana [4, 5, 6]. SSBs were found the most advertised ones on the basis of a 2022 study [7]. The highly advertised SSBs on the Ghanaian market inlcude sodas, energy drinks, bottled or canned juice drinks, coffee‐based, milk‐based and water‐based beverages [8]. In the United States, soda is one of the most consumed SSB among adults [9]. In Brazil, sodas, sweetened coffee and teas were highly consumed among adolescents, adult and the aged [10]. High prevalence of SSBs intake among the adult population has been reported in India and South Africa [11, 12]. Studies have reported on high patronage of SSBs among University students and primary school children in Ghana [4, 13]. Investigations about the consumption of SSBs in other professions in Ghana have been conducted as indicated. But the rate of consumption and most consumed SSBs among the healthcare professionals are not reported in studies from Ghana.

The pull and push factors reported to influence intake of SSBs include male gender, urbanisation, obesity, eating away from home, physical inactivity, sedentary behaviour, daily smokers, inadequate nutrition knowledge, low educational levels and socio‐economic status [9, 12, 14, 15, 16, 17]. In Ghana, taste and convenience have been reported to contribute to SSBs intake [18]. The consumption of SSBs is not without implications. In Africa, the high consumption of SSBs has been linked with high fasting blood glucose and triglyceride levels [19]. Systematic reviews and meta‐analysis showed that an increased consumption of SSB is associated with an increased rate of hypertension, weight gain, cardiovascular morbidity and cardiometabolic diseases, such as Type 2 diabetes [20, 21, 22, 23, 24, 25]. Mortality rate attributed to SSBs were 5%, 70.9% and 24.1% for low, middle and high‐income countries, respectively [26].

Due to the compelling evidence presented by different studies of the aetiology of most non‐communicable diseases (NCDs) and complications associated with the habitual consumption of SSBs, there are recommendations to governments and health care professionals to implore varied strategies, including nutrition education, taxes, public advocacy, regulation of media advertisement of SSBs and behaviour change initiatives, to discourage their usage [1, 17, 21, 27, 28, 29]. Studies have indicated that high taxes on SSBs reduced purchases, demands and consumption and also led to the reduction of weight gain [30, 31, 32]. It is also projected that high taxes on SSBs may lessen all forms of burden posited by Type 2 diabetes [33]. Among developing countries, South Africa and Mexico pioneered the introduction of excise taxes on SSBs when recommended by WHO, and in Mexico it has been reported that the introduction of the tax has contributed to significant reduction of purchases of SSBs in the population [34]. Recently, the government of Ghana also introduced excise tax on SSBs and health care professionals spearheaded the advocacy against the use of these beverages.

Do health care professionals campaigning for the zero or limited use of SSBs indulge in them or not? There is a paucity of information about the consumption of SSBs among health professionals in Northern Ghana. This study sought to assess the rate, types consumed and factors that influence consumption of SSBs among health care professionals in the northern region of Ghana.

## 2 Materials and Methods

### 2.1 Study Area, Design and Participants

The cross‐sectional study was conducted in four health facilities in the Tamale Metropolis. The Metropolis has 19 health facilities, including private and public. The facilities were randomly selected among the pool of health facilities in the Metropolis. The facilities were numbered 1–19 and folded in a box, and research assistants randomly selected four. Using the Cochran's formula for sample size calculation, with a dropout rate of 15%, a total of 345 participants were randomly recruited for the study. A probability proportionate to size sampling was used to estimate the samples for each facility from the total population and a simple random sampling was used to select the participants from each facility. The health care professionals included in the studies based on the available data from the facilities were medical doctors, physician/medical assistants, medical laboratory scientist, psychiatrist, psychologist, physiotherapist, phlebotomist, radiologist/radiographers, sonographers, nurses/midwives, nutritionist/dietitians and pharmacist/dispensary technicians. The members on the staff list of each facility were given a unique identification number, and a computer‐generated random number sequence was used to select the needed sample. All health care professionals in the selected facilities qualified to participate in the study; however, those who did not sign the inform consent were excluded from the study.

### 2.2 Data Collection

#### 2.2.1 Questionnaires

A semi‐structured questionnaire was used to collect data on sociodemographic (sex, age, marital status, ethnicity, health care profession, income level, educational level), awareness, source of information, reasons for consumption, frequency and quantities of consumption of SSBs. The questionnaire for consumption of the SSBs was adapted from Vanderlee et al. [35] and Hedrick et al. [36].

The questionnaire also included information on screen time: Period spent watching television, using computers/laptops and phones/tablets were assessed using the digital screen exposure questionnaire [37]. The questionnaire was pretested to ensure validity and reliability. The data were collected with the aid of research assistants between the period of May 2023 and July 2023.

#### 2.2.2 Statistical Analysis

The statistical package for social sciences (SPSS version 25) was used in the data analysis. Descriptive statistics was used to analyse the sociodemographic characteristics, awareness, frequency and quantity of SSBs consumption and screen time. Chi‐square was used to test the association between screen time and SSBs intake. Logistic regression was used to predict factors contributing to SSBs consumption. Data were presented in means, standard deviations, percentages and frequencies. All *p* values less than 0.05 were statistically significant.

#### 2.2.3 Ethic Statement

The study complied with Helsinki Declarations. The study protocol was reviewed and approved by the ethics review board of the University for Development Studies (UDS/RB/016/23), Ghana. Permission was granted by the Metropolitan Health Directorate of the Ghana Health Service in the Northern Region and the Health facilities that the study was conducted. The aim and benefits of the study were explained to participants. Participants who gave their approval and signed the inform consent were permitted to participate in the research.

## 3 Results

### 3.1 Sociodemographic Characteristics and Awareness of SSBs

A total of 345 health professionals participated in the study. The majority of the participants were within the age (years) range of 20–30 (54.2%) and females (69.9%) dominated. Most of the participants were married (59.4%), and the predominant health profession was nursing/midwifery (69.3%). A large proportion of the participants had diploma (44.9%) and graduate certification (47.6%). More than half (54%) of the participants earned less income, 42.3% had moderate income level, and very few participants (3.7%) earned high income (Table 1).

**TABLE 1:** Sociodemographic characteristics and awareness of sugar‐sweetened beverages.

Sociodemographic characteristics	*N* (%)
**Sex**	
Male	104 (30.1)
Female	241 (69.9)
**Age groups (years)**	
≤19	5 (1.4)
20–30	187 (54.2)
31–40	125 (36.3)
41–50	23 (6.7)
>50	5 (1.4)
**Marital status**	
Single	136 (39.4)
Married	205 (59.4)
Separated	1 (0.3)
Divorced	2 (0.6)
Widowed	1 (0.3)
**Ethnicity**	
Dagomba	148 (42.9)
Gonja	28 (8.1)
Mamprusi	26 (7.5)
Akan	32 (9.3)
Others	111 (32.2)
**Health professions**	
Medical doctor	22 (6.4)
Nurse/Midwife	239 (69.3)
Medical laboratory scientist	17 (4.9)
Pharmacist	17 (4.9)
Nutritionist	30 (8.7)
Others	20 (5.8)
**Income level (GhC)**	
<1000	53 (15.4)
1000–2000	133 (38.6)
2100–3000	107 (31)
3100–4000	39 (11.3)
>4000	13 (3.7)
**Educational level**	
SHS/Certificate in CH	26 (7.5)
Diploma	155 (44.9)
Degree	164 (47.6)

Awareness of SSBs	*N* (%)
**Heard**	
Yes	263 (76.2)
No	82 (23.8)
**Define**	
Yes	180 (68.4)
No	83 (31.6)
**Sources of information**	
Peers	71 (27)
Print media	39 (14.8)
Electronic media	44 (16.7)
Social media	70 (26.7)
Family	39 (14.8)
**Ever consumed SSBs**	
Yes	336 (97.4)
No	9 (2.6)
**Reasons for SBB intake**	
Taste	169 (50.3)
Colour	3 (0.9)
For energy	73 (21.7)
To quench thirst	58 (17.3)
Convenience	30 (8.9)
Others	3 (0.9)

*Note:* Frequency (*N*), percentage (%). Ethnicity (others—Fantes, Ewes, Guans, Frafras, Dagaabas, Konkonbas and Fulanis), health care professions (others—phlebotomist, psychiatrist, psychologist, physiotherapist). Degree (bachelors and masters) descriptive statistics.

Abbreviation: SSBs, sugar‐sweetened beverages.

A high number of the participants were aware (76.2%) of SSBs and could also define (68.4%) SSBs. The participants generally had their source of information about SSBs from the media, social (26.7%), electronic (16.7%) and print (14.8%), and peers (27%). A greater proportion (97.4%) indicated ever‐consumed SSBs, and the reason for consumption was largely for its taste (50.3%), energy (21.7%) and quenching thirst (17.3%) (Table 1).

### 3.2 Frequency and Quantity of SSBs Consumption

The combined frequency per day and week consumption of sweetened local juice (61.6% vs. 38.4%), regular soft drink (50.4% vs. 49.6%) and sweetened tea or coffee (73.6% vs. 26.4%) was higher compared to those who did not take any of these beverages at any time. Nevertheless, the frequency of those who did not (86.4%) consume sport and energy drink at any time was higher than those who took it per day and week combined (13.6%) (Table 2).

**TABLE 2:** Frequency and quantity of sugar‐sweetened beverages consumption.

Frequency	
Variable	*N* (%)
**Sweetened local juice**	
Once per day	90 (26.1)
2–3 times per day	26 (7.5)
4 or more times per day	2 (0.6)
Once per week	57 (16.5)
2–3 times per week	32 (9.3)
4 or more times per week	4 (1.2)
Do not take	134 (38.4)
**Regular soft drinks**	
Once per day	51 (14.8)
2–3 times per day	22 (6.4)
4 or more times per day	9 (2.6)
Once per week	55 (15.9)
2–3 times per week	34 (9.9)
4 or more times per week	3 (0.9)
Do not take	171 (49.6)
**Sweetened tea or coffee**	
Once per day	151 (43.8)
2–3 times per day	35 (10.1)
4 or more times per day	5 (1.4)
Once per week	27 (7.8)
2–3 times per week	24 (7.0)
4 or more times per week	12 (3.5)
Do not take	91 (26.4)
**Sport and energy drinks**	
Once per day	16 (4.6)
2–3 times per day	9 (2.6)
4 or more times per day	4 (1.2)
Once per week	12 (3.5)
2–3 times per week	2 (0.6)
4 or more times per week	4 (1.2)
Do not take	298 (86.4)

Quantity	
Variable	*N* (%)
**Sweetened local juice**	
Less than 250 mL	44 (20.9)
250 mL or 1 cup	51 (24.2)
350 mL or small bottle	96 (45.5)
500 mL or medium bottle	15 (7.1)
More than 500 mL	5 (2.4)
**Regular soft drinks**	
Less than 250 mL	22 (12.6)
250 mL or 1 cup	25 (14.4)
350 mL or small bottle	96 (55.2)
500 mL or medium bottle	26 (14.9)
More than 500 mL	5 (2.9)
**Sweetened tea or coffee**	
Less than 250 mL	60 (23.6)
250 mL or 1 cup	161 (63.4)
350 mL or small bottle	15 (5.9)
500 mL or medium bottle	15 (5.9)
More than 500 mL	3 (1.2)
**Sport and energy drinks**	
Less than 250 mL	4 (8.5)
250 mL or 1 cup	9 (19.1)
350 mL or small bottle	20 (42.6)
500 mL or medium bottle	13 (27.7)
More than 500 mL	1 (2.1)

*Note:* Frequency (*N*), percentage (%), descriptive statistics.

A large proportion of the participants consumed 350 mL or a small bottle of sweetened local juice (45.5%), regular soft drink (55.2%), sport and energy drink (42.6%) with the exception of sweetened tea/coffee having majority (63.4%) consuming 250 mL or 1 cup at any time (Table 2).

### 3.3 Screen Time of Respondents

The time spent on television, computer/laptop and phone/tablet is shown in Table 3. The proportion of participants spending 30 min to 4 or more hours watching television was 79.1% and those who did not was 20.9%. Although approximately 61% of the participants spent 30 min to 4 or more hours using computer/laptop, 39% did not spend any time at all. On the account of phone/tablet usage, 56.2% of the participants spent 4 or more hours using phone/tablet, 42.9% spent between 30 min and 3 h, and 0.9% did not (Table 3).

**TABLE 3:** Screen time of respondents.

Variable	Frequency	Percentage
**Television**		
Zero	72	20.9
30 min	85	24.6
1 h	71	20.6
2 h	41	11.9
3 h	28	8.1
4 or more hours	48	13.9
**Computer or laptop**		
Zero	136	39.4
30 min	49	14.2
1 h	64	18.6
2 h	32	9.3
3 h	27	7.8
4 or more hours	37	10.7
**Phone or tablet**		
Zero	3	0.9
30 min	36	10.4
1 h	34	9.9
2 h	41	11.9
3 h	37	10.7
4 or more hours	194	56.2

*Note:* Descriptive statistics.

### 3.4 Association Between Screen Time and SSBs Consumption

The results showed significant association between time spent on using phone/tablet and sweetened local juice/beverages intake (*X* ^2^ = 49.12, *p* = 0.02). Time spent watching television was significantly associated with an intake of regular soft drink (*X* ^2^ = 58.89, *p* = 0.001), sweetened coffee/tea (*X* ^2^ = 45.01, *p* = 0.04) and sport and energy drinks (*X* ^2^ = 71.19, *p* < 0.001), respectively (Table 4).

**TABLE 4:** Association between screen time and sugar‐sweetened beverages consumption.

Screen time	Sweetened local juice/beverage intake								Regular soft drink consumption							
	Once per day	2–3 times per day	4 or more times per day	Once per week	2–3 times per week	4 or more times per week	Don't take	Test statistic	Once per day	2–3 times per day	4 or more times per day	Once per week	2–3 times per week	4 or more times per week	Don't take	Test statistic
	*N* (%)	*N* (%)	*N* (%)	*N* (%)	*N* (%)	*N* (%)	*N* (%)	***X* ^2^ *P***	*N* (%)	*N* (%)	*N* (%)	*N* (%)	*N* (%)	*N* (%)	*N* (%)	***X* ^2^ *P***
**TV**																
Zero	16 (17.8)	4 (15.4)	0 (0)	19 (33.3)	9 (28.1)	0 (0)	24 (17.9)		8 (15.7)	7 (31.8)	0 (0)	13 (23.6)	5 (14.7)	0 (0)	39 (22.8)	
30 min	29 (32.2)	7 (26.9)	0 (0)	9 (15.8)	7 (21.9)	0 (0)	33 (24.6)		12 (23.5)	7 (31.8)	0 (0)	9 (16.4)	7 (20.6)	1 (33.3)	49 (28.7)	
1 h	20 (22.2)	4 (15.4)	2 (100)	11 (19.3)	7 (21.9)	2 (50)	25 (18.7)		7 (13.7)	2 (9.1)	8 (88.9)	14 (25.5)	8 (23.5)	2 (66.7)	30 (17.5)	
2 h	6 (6.7)	4 (15.4)	0 (0)	6 (10.5)	6 (18.8)	1 (25)	18 (13.4)	**37.14 0.17**	7 (13.7)	3 (13.6)	1 (11.1)	4 (7.3)	7 (20.6)	0 (0)	19 (11.9)	**58.89 0.001**
3 h	4 (4.4)	2 (7.7)	0 (0)	7 (12.3)	0 (0)	0 (0)	15 (11.2)		5 (9.8)	3 (13.6)	0 (0)	3 (5.5)	1 (2.9)	0 (0)	16 (9.4)	
4 or more hours	15 (16.7)	5 (19.2)	0(0)	5 (8.8)	3 (9.4)	1 (25)	19 (14.2)		12 (23.5)	0 (0)	0 (0)	12 (21.9)	6 (17.6)	0 (0)	18 (10.5)	
**Computer/Laptop**																
Zero	46 (51.1)	6 (23.1)	2 (100)	21 (36.8)	7 (21.9)	0 (0)	54 (40.3)		18 (35.3)	3 (13.6)	2 (22.2)	25 (45.5)	6 (17.6)	0 (0)	82 (48.0)	
30 min	8 (8.9)	4 (15.4)	0 (0)	12 (21.1)	7 (21.9)	1 (25)	17 (12.7)		6 (11.8)	5 (22.7)	0 (0)	7 (12.7)	8 (23.5)	1 (33.3)	22 (12.9)	
1 h	13 (14.4)	6 (23.1)	0 (0)	9 (15.8)	9 (28.1)	1 (25)	26 (19.4)		10 (19.6)	7 (31.8)	2 (22.2)	10 (18.2)	8 (23.5)	1 (33.3)	26 (15.2)	
2 h	8 (8.9)	5 (19.2)	0 (00	6 (10.5)	0 (0)	1 (25)	12 (9)	**35.30 0.23**	5 (9.8)	3 (13.6)	1 (11.1)	5 (9.1)	6 (17.6)	0 (0)	12 (7)	38.11 0.15
3 h	9 (10)	1 (3.8)	0 (0)	4 (7)	3 (9.4)	1 (25)	9 (6.7)		5 (9.8)	2 (9.1)	2 (22.2)	5 (9.1)	4 (11.8)	0 (0)	9 (5.3)	
4 or more hours	6 (6.7)	4 (15.4)	0 (0)	5 (8.8)	6 (18.8)	0 (0)	16 (11.9)		7 (13.7)	2 (9.1)	0 (0)	3 (5.5)	2 (5.9)	1(33.3)	20 (11.7)	
**Phone/Tablet**																
Zero	0 (0)	0 (0)	0 (0)	3 (5.3)	0 (0)	0 (0)	0 (0)		0 (0)	0 (0)	0 (0)	1 (1.8)	0 (0)	0 (0)	2 (1.2)	
30 min	8 (8.9)	1 (3.8)	0 (0)	4 (7)	1 (3.1)	0 (0)	22 (16.4)		4 (7.8)	1 (4.5)	0 (0)	4 (7.3)	1 (2.9)	0 (0)	26 (15.2)	
1 h	16 (17.8)	1 (3.8)	0 (0)	4 (7)	3 (9.4)	0 (0)	10 (7.5)		4 (7.8)	1 (4.5)	0 (0)	5 (9.1)	3 (8.8)	0 (0)	21 (12.3)	
2 h	11 (12.2)	2 (7.7)	0 (0)	6 (10.5)	2 (6.2)	0 (0)	20 (14.9)	**49.12 0.02**	9 (17.6)	3 (13.6)	4 (44.4)	5 (9.1)	2 (5.9)	0 (0)	18 (10.5)	32.06 0.37
3 h	8 (8.9)	2 (7.7)	0(0)	8 (14)	2 (6.2)	2 (50)	15 (11.2)		6 (11.8)	4 (18.2)	0 (0)	8 (14.5)	3 (8.8)	0 (0)	16 (9.4)	
4 or more hours	47 (52.2)	20 (76.9)	2 (100)	32 (56.1)	24 (75)	2 (50)	67 (50)		28 (54.9)	13 (59.1)	5 (55.6)	32 (58.2)	25 (73.5)	3 (100)	88 (51.5)	
Screen time	Sweetened tea/coffee								Sport and energy drink consumption							
	*N* (%)	*N* (%)	*N* (%)	*N* (%)	*N* (%)	*N* (%)	*N* (%)	***X* ^2^ *P***	*N* (%)	*N* (%)	*N* (%)	*N* (%)	*N* (%)	*N* (%)	*N* (%)	***X* ^2^ *P***
**TV**																
Zero	26 (17.2)	8 (22.9)	0 (0)	6 (22.2)	2 (8.3)	1 (8.3)	28 (31.9)		4 (25)	0 (0)	0 (0)	3 (25)	0 (0)	0 (0)	65 (21.8)	
30 min	37 (24.5)	7 (20)	0 (0)	5 (18.5)	11 (45.8)	4 (33.3)	21 (23.1)		3 (18.8)	5 (55.6)	0 (0)	1 (8.3)	0 (0)	0 (0)	76 (25.5)	
1 h	34 (22.5)	4 (11.4)	2 (40)	7 (25.9)	5 (20.8)	3 (25)	16 (17.6)		2 (12.5)	2 (22.2)	1 (25)	7 (58.3)	0 (0)	3 (75)	56 (18.8)	
2 h	18 (11.9)	9 (25.7)	1 (20)	1 (3.7)	2 (8.3)	3 (25)	7 (7.7)	**45.01 0.04**	2 (12.5)	2 (22.2)	3 (75)	0 (0)	0 (0)	0 (0)	34 (11.4)	**71.19** < **0.001**
3 h	11 (7.3)	0 (0)	0 (0)	4 (14.8)	2 (8.3)	1 (8.3)	10 (11)		1 (6.2)	0 (0)	0 (0)	0 (0)	2 (100)	0 (0)	25 (8.4)	
4 or more hours	25 (16.6)	7 (20)	2 (40)	4 (14.8)	2 (8.3)	0 (0)	8 (8.8)		4 (25.6)	0 (0)	0 (0)	1 (8.3)	0 (0)	1 (25)	42 (14.1)	
**Computer/Laptop**																
Zero	58 (38.4)	11 (31.4)	2 (40)	8 (29.6)	12 (50)	2 (16.7)	43 (47.3)		4 (25)	3 (33.3)	0 (0)	5 (41.7)	2 (100)	0 (0)	122 (40.9)	
30 min	25 (16.6)	2 (5.7)	0 (0)	9 (33.3)	1 (4.2)	2 (16.7)	10 (11.0)		3 (18.8)	2 (22.2)	2 (50)	0 (0)	0 (0)	0 (0)	42 (14.1)	
1 h	26 (17.2)	8 (22.9)	0 (0)	6 (22.2)	2 (8.3)	3 (25)	19 (20.9)		1 (6.2)	2 (22.2)	0 (0)	4 (33.3)	0 (0)	2 (50)	55 (18.5)	
2 h	10 (6.6)	4 (11.4)	2 (40)	4 (14.8)	4 (16.7)	1 (8.3)	7 (7.7)	**42.26 0.07**	3 (18.8)	0 (0)	1 (25)	0 (0)	0 (0)	1 (25)	27 (9.1)	37.50 0.16
3 h	15 (9.9)	4 (11.4)	0 (0)	0 (0)	2 (8.3)	1 (8.3)	5 (5.5)		3 (18.8)	2 (22.2)	0 (0)	0 (0)	0 (0)	1 (25)	21 (7)	
4 or more hours	17 (11.3)	6 (17.1)	1 (20)	0 (0)	3 (12.5)	3 (25)	7 (7.7)		2 (12.5)	0 (0)	1 (25)	3 (25)	0 (0)	0 (0)	31 (10.4)	
**Phone/Tablet**																
Zero	1 (0.7)	0 (0)	0 (0)	0 (0)	0 (0)	0 (0)	2 (2.2)		0 (0)	0 (0)	0 (0)	0 (0)	0 (0)	0 (0)	3 (1)	
30 min	19 (12.6)	1 (2.9)	2 (40)	1 (3.7)	1 (4.2)	0 (0)	12 (13.2)		1 (6.2)	0 (0)	0 (0)	0 (0)	2 (100)	0 (0)	33 (11.1)	
1 h	16 (10.6)	2 (5.7)	0 (0)	5 (18.5)	2 (8.3)	0 (0)	9 (9.9)		0 (0)	1 (11.1)	0 (0)	0 (0)	0 (0)	2 (50)	31 (10.4)	
2 h	24 (15.9)	3 (8.6)	0 (0)	3 (11.1)	4 (16.7)	0 (0)	7 (7.7)	**34.22 0.27**	2 (12.5)	2 (22.2)	0 (0)	4 (33.3)	0 (0)	0 (0)	33 (11.1)	**44.01 0.05**
3 h	15 (9.9)	5 (14.3)	1 (20)	4 (14.8)	0 (0)	1 (8.3)	11 (12.1)		2 (12.5)	2 (22.2)	0 (0)	0 ()	0 (0)	1 (25)	32 (10.7)	
4 or more hours	76 (50.3)	24 (68.6)	2 (40)	14 (51.9)	17 (70.8)	11 (91.7)	50 (54.9)		11 (68.8)	4 (44.4)	4 (100)	8 (66.7)	0 (0)	1 (25)	166 (55.7)	

*Note:* Frequency (*N*), percentage (%), chi‐square test (*X* ^2^), *p* < 0.05.

### 3.5 Predictors of SSBs Consumption

The people with Akan background were less likely to consume sweetened local juice (OR = 0.29, *p* = 0.02, 95% CI: 0.10–0.81). The odds of consuming sweetened local juice was higher among nurses (OR = 11.42, *p* < 0.05, 95% CI: 2.55–51.22), pharmacist (OR = 6.08, *p* = 0.04, 95% CI: 1.07–34.58) and nutritionist (OR = 5.37, *p* = 0.04, 95% CI: 1.02–28.31). The married participants had higher odds of consuming regular soft drinks (OR = 3.27, *p* < 0.05, 95% CI: 1.72–6.21). The outcome showed that irrespective of the period, watching television was protective against the consumption of sweetened tea/coffee. The female gender (OR = 4.07, *p* < 0.05, 95% CI: 1.79–9.26) and participants having spouse (OR = 3.61, *p* < 0.05, 95% CI: 1.40–9.32) had higher risk of consuming sport and energy drink (Table 5).

**TABLE 5:** Predictors of sugar‐sweetened beverages consumption.

Type of SSB	Wild	Sig	Exp. (*B*)	95% CI
**Sweetened local juice**				
Ethnicity (reference: Dagomba)	10.706	**0.03**		
Gonja	0.543	0.46	0.71	0.28–1.79
Mamprusi	3.258	0.07	0.37	0.12–1.09
Akan	5.570	**0.02**	0.29	0.10–0.81
Others	0.384	0.54	1.20	0.68–2.12
Health professions (reference: Med. doctor)	12.874	**0.03**		
Nurse/Midwife	10.118	0.001	11.42	2.55–51.22
Med. laboratory scientist	3.643	0.05	5.76	0.95–34.83
Pharmacist	4.138	0.04	6.08	1.07–34.58
Nutritionist	3.924	0.04	5.37	1.02–28.31
Others	3.778	0.05	6.20	0.99–39.07
Constant	0.000	0.99	0.00	
**Regular soft drink**				
Marital status (reference: single)	13.148	**0.01**		
Married	13.059	<0.001	3.27	1.72–6.21
Separated	0.000	1.000	8,728,795,039	0.000
Divorced	0.291	0.590	2.30	0.11–47.27
Widowed	0.000	1.000	2,519,887,494	0.000
Educational level (reference: SHS/certificate)	6.439	**0.04**		
Diploma	3.820	0.05	2.58	1.00–6.67
Degree	0.523	0.45	1.48	0.53–4.13
Constant	0.081	0.78	0.76	
**Sweetened tea/coffee**				
TV time (reference: zero)	14.117	**0.02**		
30 min	6.034	0.01	0.39	0.19–0.83
1 h	6.557	0.01	0.35	0.16–0.78
2 h	6.250	0.01	0.28	0.10–0.76
3 h	0.056	0.81	0.88	0.32–2.45
4 or more hours	5.994	0.01	0.31	0.12–0.79
Constant	2.543	0.11	0.14	
**Sport and energy drink**				
Gender (female)	11.196	**0.001**	4.07	1.79–9.26
Marital status (reference: single)	10.525	**0.03**		
Married	7.049	0.008	3.61	1.40–9.32
Separated	0.000	1.000	1,83,550,036.2	0.000
Divorced	1.003	0.317	0.19	0.01–4.80
Widowed	0.000	1.000	1,63,793,446.3	0.000
Constant	0.000	0.999	4,51,912,576.6	

*Note:* Logistics regression, *p* < 0.05.

## 4 Discussion

SSBs are implicated in the aetiology of NCDs globally [19, 20, 23, 38]. To prevent and reduce the burden of NCDs in populations, it is important to identify the key dietary influencers, such as intake and predictive factors of SSBs. The aim of the study was to assess the predictors and prevalence of SSBs consumption among health professionals in the Tamale Metropolis.

The current findings revealed that the majority of the participants were aware and could also explain SSBs. An outcome from a study in Australia showed otherwise [39]. This may be due to the professions of the study participants. The outcome showed that messages about SSBs were received largely from the media (print, electronic and social) and peers. This outcome agrees with a review report that summarised that media was profound in informing participants of SSBs [40].

Our findings revealed high prevalence of SSBs consumption. This shows that their high knowledge in SSBs has little or no influence on their consumption of SSBs, suggesting behaviour gap among health care professionals in SSBs consumption. Besides, it appears that the introduction of excise tax on these products in Ghana is not deterrent enough to reduce purchase of these products. This outcome adds to reports from other studies in different countries, such as India and South Africa, indicating a high consumption of SSBs in the adult population [11, 12, 20]. The results indicated that sweetened local juice, regular soft drink and sweetened coffee/tea were the most frequently consumed SSBs. This outcome is similar to that reported among adults in Brazil [10]. The quantity of SSBs that were mostly consumed in the present study at each point was 350 mL or small bottle for sweetened local juice, regular soft drink and sport and energy drink. Nevertheless, sweetened tea/coffee had a least consumption of 250 mL or 1 cup at any time. The quantity of intake SSBs by our study participants was higher compared to that reported by Subramanian et al. [11] among the adult population (100–200 mL) in India. Taste being the main reason for SSBs intake in the present study, the same was given by the study participants in India.

The present study showed that the time spent on television significantly influenced the consumption of regular soft drink, sweetened tea/coffee and sports and energy drinks. A study showed that these SSBs are the most advertised in many television stations in Ghana [8], and this might have contributed to the regular consumption by the study participants. Furthermore, the food environment in the hospital may have also been flooded with these products, which may have also influenced their intake due to availability and accessibility by the health care practitioners. This finding concurs with studies in India and the United Kingdom that reported similar outcome [41, 42]. Moreover, the use of phones or tablets influenced the consumption of sweetened local juice/beverages.

The results revealed that participants in the health care professions, such as nurses, pharmacists and nutritionists, had a higher risk of consuming sweetened local juice. The high consumption of the local beverages may be due to the minimal technology in processing and the use of no artificial flavours or additives in preserving them by the indigenous people. Moreover, some of the ingredients used in the preparation of the local juices are perceived locally to be healthy and medicinal, hence the increase in consumption. Married couples were more likely to consume regular soft drinks and sport and energy drinks. The female participants had higher odds of consuming sports and energy drinks. These outcomes were consistent with studies from India [41] and Malaysia [43] that reported that marital status and sex influenced the consumption of aerated drinks. However, in Australia, United Kingdom and Saudi Arabia, males were more likely to consume these SSBs [16, 42, 44]. Furthermore, the results showed that any time spent on watching television reduced the odds of sweetened tea/coffee intake. This was contrary to a study in Saudi Arabia that showed sitting behind television increased the risk of SSBs consumption [44].

### 4.1 Practical Applications

To address the high consumption of SSBs, it appears that the excise tax alone is not enough to reduce the consumption, we encourage that government should introduce or increase taxes on advertisement on these products in the media and also pass laws that would permit the advertisement to be shown at odd hours of the day. The government should consistently increase the excise tax on SSBs. In addition, the Food and Drugs Authority should enact laws and enforce manufacturers to indicate the negative implications (with pictures or images) of consumption of these SSBs as part of the nutrition information on the products. In addition, the Ministry of Health should as a matter of urgency promote healthy food environment in the cafeteria, canteens and restaurants in the hospitals. We further recommend that researchers investigate the impact of hospital food environment on the health of health care professionals and patients.

### 4.2 Strength and Limitations

The study provides insight into frequency and quantity of SSBs consumed and its precipitating factors among health care professionals. As a limitation to this study, causal relationship could not be established due to the study design used. Again, recall bias could affect the reportage of the SSBs consumption.

## 5 Conclusion

The participants were largely aware of SSBs, and the media in any form was the easiest means of receiving information about SSBs. There was high prevalence of SSB intake, and the frequently used ones were sweetened local juice, regular soft drink and sweetened tea or coffee, and quantity of intake ranging from one cup to small bottle. The period spent on watching television and using phones or tablets influenced the consumption of SSBs. Health professionals consumed more of sweetened local juice. The results of this research can help policymakers build an enhanced intervention approach to reduce added sugar consumption among health professionals. The Ministry of Health should promote behaviour change and award professionals with none or low intake of SSBs as well as support policy and promote the sales of healthy foods at health facilities to purge the food environment of SSBs.

## Author Contributions

**Michael Akenteng Wiafe**: conceptualisation, writing – original draft, investigation, writing – review and editing, methodology, formal analysis, supervision, data curation. **Hamza Sulemana**: conceptualisation, investigation, writing – original draft, methodology, validation, formal analysis, data curation, software. **Gabriel Libienuo Sowley Kalog**: conceptualisation, writing – original draft, writing – review and editing, visualization, methodology, formal analysis, supervision. **Kwadwo Densi Amankwah**: conceptualisation, writing – original draft, methodology, software, data curation, project administration.

## Funding

The authors have nothing to report.

## Conflicts of Interest

The authors declare no conflicts of interest.

## Data Availability

Upon reasonable request, data for this study can be made available by writing to the Chairman, Committee on Human Research Publication Ethics, ethics review board of University for Development Studies, P.O. Box TL 1350, Tamale, Ghana.
